# Medical Student Interpretation of Visual Art: Who's Got Empathy?

**DOI:** 10.15694/mep.2018.0000206.2

**Published:** 2018-10-17

**Authors:** Samuel Sampson, Johanna Shapiro, John Boker, Joel Shallit, Julie Youm, John Billimek

**Affiliations:** 1 Kaiser Permanente Los Angeles Medical Center; 2 University of California Irvine School of Medicine

**Keywords:** arts in medicine, medical student empathy, empathy measurement, medical humanities

## Abstract

This article was migrated. The article was marked as recommended.

**Introduction:** Physician empathy is a highly desired characteristic in clinical practice with benefits for both patients and doctors. Increasingly, medical educators have acknowledged the importance of empathy and sought effective ways of inculcating and strengthening this quality in medical students. However, empathy remains difficult to measure because of differing definitions and theoretical dimensions. Our goal was to develop a de novo visual Art scale, devised to evaluate empathetic response in medical students as well as a de novo Biosocial scale to measure medical student socioeconomic and experiential stress during childhood and adolescence; and to compare these exploratory measures to the reliable and well-validated Jefferson Scale of Empathy JSE).

**Methods:** We constructed a survey incorporating a visual Art empathy measure, a Biosocial scale, and the JSE, which we sent to approximately 200 allopathic preclinical medical students at our home institution. We received 71 complete responses.

**Results:** Cronbach’s alpha testing found that the items in both new scales had adequate reliability. Multivariate regression analysis found a significant, positive association between both the visual art and biosocial scores and the JSE.

**Discussion:** These results support the idea that response to visual stimuli, as well as life stressors, may be factors in medical students’ capacity to formulate an empathetic response to patients.

## Introduction

Mounting evidence points to the conclusion that physicians expressing empathy toward patients and families is not only “the right thing to do” from a moral perspective, but has multiple benefits for patients and physicians. This body of research has resulted in greater attention to methods for assessing and training empathy in medical students and residents. Empathy awareness and skill-building takes many forms, including exposure to humanities and arts. Disagreements persist about whether it is possible for physicians to be too empathetic, and even about the definition of empathy itself.

While still controversial, in the future personal attributes such as empathy may also emerge as factors in selecting applicants for medical school. If so, it is likely that admissions committees will seek valid, reliable instruments to assess such qualities. The Jefferson Scale of Empathy is a leading instrument for assessing clinical empathy in health professionals, with well-established reliability and validity and a large and robust body of research documenting empathy levels and changes in empathy in a wide variety of healthcare populations. For both theoretical and psychometric reasons, it assesses primarily cognitive dimensions of empathy. While an invaluable tool, it may not fully elicit important dimensions of empathy, which potentially could be assessed in other ways. The goal of this study was to begin to explore these other dimensions.

**Effects of physician empathy on patients.** Patients who have empathetic physicians are more compliant with their treatments ( Kim, Kaplowitz and Johnston, 2004), are more satisfied with their care ( Hojat *et al.,* 2011a; Derksen, Bensing and Lagro-Janssen, 2013), and have better health outcomes ( Hojat *et al.,*2011b; Del Canale *et al.,* 2012). When doctors demonstrate empathy, patients are less anxious ( Fogarty *et al.,* 1999), and diagnostic accuracy is improved, leading to better treatment planning and patient adherence to treatment regimen ( Zolnierek, 2009; Hojat *et al*., 2011b). One study of surgical outcomes concluded that surgeon empathy had a significantly positive indirect effect on patient satisfaction through its relationship to patients’ self-reported health status ( Weng, 2011) **;** while another study concluded that physician empathy predicted patients’ perceived medical outcomes ( Steinhausen *et al.,* 2014). Other research found that patients with diabetes who perceive their physicians as empathetic have lower HA1C scores, improved low-density lipoprotein ( Hojat *et al.,*2011b), and fewer acute metabolic complications ( Del Canale *et al*., 2012), as well as improved self-management skills ( Sultan *et al.,* 2011). Patients with empathic physicians also have better immune function and recover from viral infections more quickly ( Rakel *et al*., 2009); while migraine sufferers with empathic physicians experience reduced disability( Attar and Chandramani, 2012). A recent study indicated that empathy is related to positive personality characteristics essential for building a successful doctor-patient relationship ( Hojat *et al*., 2015).

**Effects of empathy on physicians and medical students**. Empathy appears to exert a positive influence on physicians themselves ( Kelm *et al.,* 2014), and is identified as a protective factor in burnout ( Thomas *et al.,* 2007; Thirieux, Birault and Jaafari, 2016). Empathy is associated with personal accomplishment and job satisfaction for physicians ( Robieux *et al*., 2018) **,** which in the reverse are both components of burn-out. In one large survey, 87% of physicians who report erosion in their enthusiasm for medicine attribute this loss to the inhibition of empathic care ( Zuger, 2004). Empathy has also been linked to higher physician well-being ( Shanafelt *et al.,* 2005; Paro *et al.,* 2014), higher ratings of medical student clinical competence ( Hojat *et al.,*2002b), and less medical-legal risk ( Moore, Adler and Robertson, 2000; Smith *et al.,* 2016).

However, empathy in physicians can be a double-edged sword, in some cases associated with emotional exhaustion, especially in women physicians ( Gleichberrcht and Decety, 2013; Paro *et al*., 2014), distress ( DiLalla, Hull and Dorsey, 2004), compassion fatigue ( Decety, Yang and Cheng, 2010; Figley, 2012), and burnout ( Nielson and Tulinius, 2009; Zenasni *et al*., 2012), precisely those conditions which other studies show it to be protective against. The key to appropriate empathy appears to lie in skills of emotional self-regulation. Physicians who have difficulty regulating their negative arousal and describing and identifying emotions seem to be more prone to emotional exhaustion, detachment, and a low sense of accomplishment ( Gleichberrcht and Decety, 2012).

**Empathy training in medical education.** Because empathy is such an important quality for physicians, medical schools have shown increasing interest in pedagogical approaches to training empathy. Teaching empathy has been a focus in medical education for more than 20 years ( Poole and Sanson-Fisher, 1980; Feigny, Monaco and Arnold, 1995; Suchman *et al.,* 1997). Early studies showed positive effects of empathy training ( Poole and Sanson-Fisher, 1980; Feigny, Monaco and Arnold, 1995) and overall that finding has held up over time ( DiLalla, Hull and Dorsey, 2004). Two systematic reviews of empathy-enhancing interventions, including exposure to humanities and arts as well as communication skills training, concluded that most efforts were successful in improving physician and medical student empathy ( Kelm *et al*., 2014; Batt-Rawden *et al*., 2013). A landmark study used mindfulness based stress reduction training with primary care physicians that resulted in significant empathy gains ( Krasner *et al.,* 2009). A more recent systematic review concluded that mindfulness training can reduce depression and anxiety and increase resilience and empathy in health professional students ( McConville, McAleer and Hahne, 2017). Virtual patients have been used in teaching empathy ( Kleinsmith *et al*., 2015) and a recent randomized, controlled trial demonstrated that an intervention group using standardized patients showed significantly higher levels of empathy than the control group ( Wundrich *et al.,* 2017). Riess et al pioneered a behaviorally based system of empathy training and applied it to difficult clinical encounters and breaking bad news, finding significant elevations in based on patient ratings for residents from various specialties exposed to their system ( Riess *et al.,* 2011; Riess *et al.,* 2012).

**Medical school admissions and empathy.** From an admission committee perspective, selecting for desired traits such as empathy, as well as associated characteristics, might help stratify the highly-qualified pool of US medical school applicants ( Monroe *et al.,* 2013). Hojat (2014) notes that while there seems to be hesitation among U.S. leaders in medical education to use personality assessments for selection purposes, incorporating such measures in admission procedures could actually lead to more optimal and compassionate healthcare. A different approach, the Multiple Mini Interview, was pioneered at McMaster University in its application process as a more reliable method of integrating a test for empathy with assessment of other non-cognitive skills ( Weir *et al.,* 2015).

**Measuring empathy.** A widely used scale to test self-reported empathetic response in physicians, medical students, and health professionals is the Jefferson Scale of Empathy (JSE) ( Hojat *et al*., 2002a; Hojat, 2016). For both theoretical and psychometric purposes, this scale focuses on empathy as a cognitive attribute that involves an ability to understand the patient’s inner experiences and perspective and a capability to communicate this understanding. This definition intentionally excludes the more affective dimensions of empathy, which Hojat argues are more similar to sympathy, and therefore more likely to lead to difficulty with emotional regulation and consequent affective imbalance. This purposeful restriction raises the possibility that other dimensions of empathy relevant to patient care may exist that the JSE does not assess. Other theoretical models, for example, distinguish between detached and affective empathy, positing the latter as a “higher” level of empathic engagement ( Post *et al.,* 2014). Further, it is also possible that a test which utilizes written depictions of scenarios may lend itself to social desirability bias, especially amongst highly motivated students who have demonstrated excellence at test-taking.

**Art exposure and medical student empathy.** While exposure to visual art is more often used to promote visual thinking strategies and improve clinical observation skills in medical students( Klugman, Peel and Beckmann-Mendez, 2011), limited evidence exists that viewing and creating art is associated with increased empathy as well ( Katz and Khoshbin, 2014). One early qualitative study ( Shapiro, Rucker and Beck, 2006) concluded that close viewing and interpretation of art was associated with increases in medical student empathic perception. A recent review points to the value of incorporating the arts in medical education, especially for teaching self-awareness, openness to other perspectives, and empathy ( Haidet *et al.,* 2016). Another study of 3 ^rd^ year University of Hong Kong medical students using the JSE as a pre-post measure found that, while the level of empathy declined in both groups over time, with no statistically significant differences between the intervention and control groups, the qualitative data showed that students in an arts-making condition gained insight into self, patients, pain and suffering, and the role of the physician ( Potash *et al.,* 2014).

This finding is similar to conclusions of another qualitative study ( Jones, Kittendorf and Kumagai, 2017) which saw students who viewed art reflecting on the human dimensions of illness and medical care. Another study examining a variety of medical humanities and arts-based coursework, including visual art, found correlations between this exposure and superior empathy outcomes among medical students ( Graham *et al.,* 2016). Yang and Yang (2013), however, found that a 4-hour experience with interpreting paintings did not influence medical students’ scores on the JSE. While not definitive, these findings hint that there may be a significant visual element to evoking an empathetic response towards a patient. A vision-based scale of empathy may also be less vulnerable to social desirability bias.

**Physician life experience and empathy.** In addition to asking whether art can be used not only to evoke empathy but to assess empathy, we wondered about other contributors to empathy that might not be measured in current instruments. In particular we were interested in whether medical students’ formative socioeconomic factors and stressful life experiences might also be associated with empathetic response. While we found very little research on this topic pertaining specifically to physicians, one study did establish that medical students from lower socioeconomic backgrounds had more positive attitudes toward the importance of physician empathy than those from higher SES backgrounds ( van Ryn *et al.,* 2014).

This was consistent with a larger body of research indicating that, despite lack of resources and personal hardship, lower social class individuals express more empathic accuracy ( Kraus, Côté and Keltner, 2010) than individuals higher up on the socioeconomic ladder. A series of studies found that, relative to their upper-class counterparts, lower-class individuals reported elevated dispositional compassion as well as greater self-reported compassion during a compassion-inducing video and for another person during a social interaction. This report concluded that lower-class individuals are more attuned to others’ distress, relative to their upper-class counterparts ( Stellar *et al*., 2012). Another series of studies found that lower class individuals proved to be more generous, charitable, trusting, and helpful compared with their upper class counterparts. Mediator and moderator data showed that lower class individuals acted in a more prosocial fashion because of a greater commitment to egalitarian values and feelings of compassion ( Piff *et al.,* 2010).

From a theoretical perspective, scholars suggest that difficult personal experiences can be a valuable tool in cultivating empathy ( Davis, 2009), hypothesizing that personal suffering may lead to a desire to ease the suffering of others, although the evidence to support this theory is limited ( Conchar and Reppar, 2014). One empirical study found that personal experience predicted empathic understanding and prosocial behavior toward individuals with depression, but not for individuals experiencing grief ( Koopman, 2015), suggesting that personal experience may have a complex relationship to patients’ psychosocial states. Other research suggests that difficult life experiences may in fact predispose individuals to evaluate another’s difficulty in coping with a similar situation more harshly ( Ruttan, McDonnell and Nordgren, 2015).

While research increasingly suggests the value of empathy for both patients and physicians, we need to know more about which components of empathy are determinative of these positive outcomes. This gap in knowledge suggests the importance of investigating new ways of assessing empathy in medical learners. As well, understanding more about factors associated with empathy such as visual responsiveness and early stressful personal experience, may play a role in the selection of future physicians.

## Methods

**Survey administration.** After obtaining IRB approval (HS #2015-2115), we distributed the study recruitment letter electronically via email invitation to approximately 200 medical students at a U.S. public university school of medicine in (preclinical) years 1 and 2. Positive response indicated consent to participate. Subject participation took the form of answering a survey, electronically administered via suveymonkey.com. Participants were limited to one survey access per unique IP address. Although participants could choose to not submit at any time, or to leave the survey as incomplete, submitting the survey as complete required respondents to answer all survey questions.

**Biosocial scale.** The survey first collected information on socioeconomic status and personal background. These questions asked for age, gender, early family income, family financial difficulties, previous dead-end employment, history of traumatic experiences, exposure to suffering, and any history of significant personal loss. All question response options, except for gender, were ranges rather than discreet values so as to protect respondent anonymity.

For all questions except age and sex, we assigned a 1-4 point scale to each of these responses, based on which quartile of response options into which each respondent’s choice fell. We assigned a higher point value to responses we assumed to be more indicative of life stress (i.e., more traumatic experiences or a lower family income). We then calculated a cumulative “biosocial score” for each respondent by summing the point values of the responses to these six questions. The minimum biosocial score possible for each respondent was 6 points, and the maximum, 24 points.

**Jefferson Scale of Empathy.** Following the biosocial questions, the survey administered the JSE. The JSE acted as a well-studied and validated comparison study with which to correlate student empathetic response to the art portion of the study, as well as to the SES and personal history questions. Scoring of survey results was conducted in accordance with the JSE scoring system ( Hojat, Gonnella and Maxwell, 2009). As the JSE uses a 7-point Likert scale with 20 questions, the minimum score possible for each respondent was 20 points and the maximum was 140 points.

**Figure 1.**
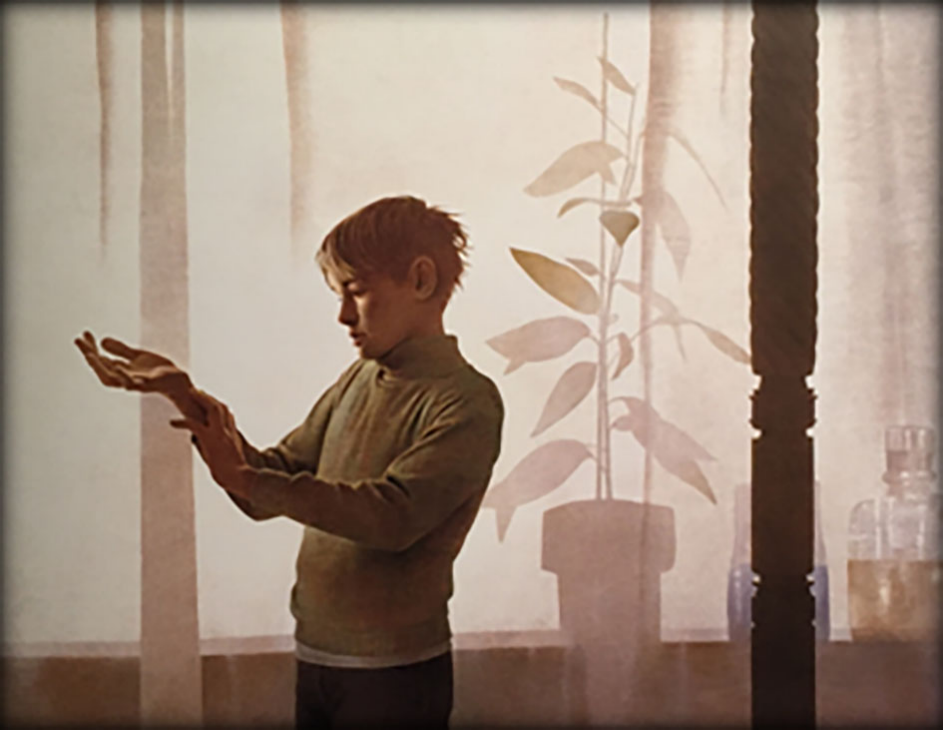
An Example of JAMA Cover Art

**Figure 2.**
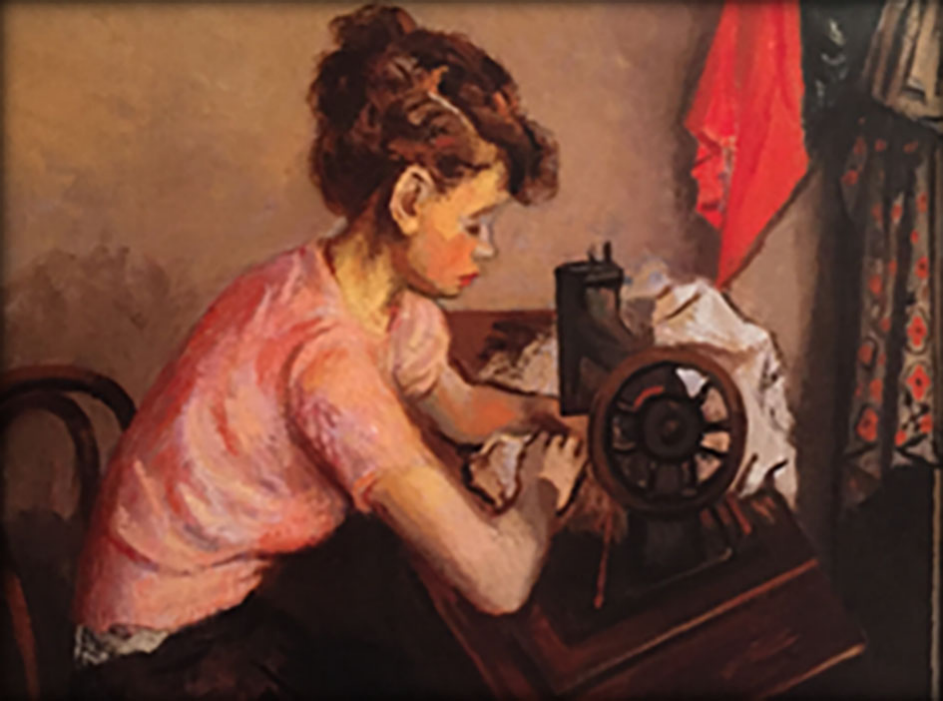
An Example of JAMA Cover Art

**Art and empathy survey.** Finally, subjects responded to the art survey component. The first author, working with a faculty mentor, selected 10 paintings for use in the study: These consisted of classic paintings in the public realm, displaying a prominent human subject, either a single individual or the central figure in a group, which also had been selected as cover art on the *Journal of the American Medical Association*, and thus judged by physicians and art enthusiasts to be relevant to humanistic- and medicine-centric ideas. Selections were balanced for sex and age of primary subject and to some extent ethnic/racial/socioeconomic diversity. Subjects first viewed each painting individually (see Figs 1, 2).

They then read six statements about the painting (see Table 1). The respondent indicated agreement with the statement on a 7-point Likert scale. Respondents had no time limit per painting and question set. All statements were identical for each painting.

**Table 1.** Visual Art Scale Statements to be Answered for each Image

1. This painting interests me.
2. I understand the painting. (reverse scale) (removed on reliability testing)
3. I am moved by the painting.
4. The painting makes me uncomfortable. (reverse scale) (removed on reliability testing)
5. I would like to think about the painting more.
6. The painting depicts a simple theme. (reverse scale)

The rationale for these items in terms of empathy was as follows: *Item 1* was intended to measure the overall “gestalt” feeling of each respondent on viewing a human subject, an expression of curiosity or interest. *Item 2* (removed to improve internal reliability) assessed the level of quick “dismissive” judgment of the respondent, to contrast with a desire to learn more. *Item 3* determined the respondent’s emotional response in relation to the subject’s situation and/or emotions. *Item 4* (removed) assessed the tolerance of the respondent to ambiguous, emotionally charged depictions. *Item 5* assessed the respondent’s curiosity and willingness to further explore the human context. *Item 6* measured an understanding of complexity and subtleties. As there were 10 paintings with 4 questions each, and a 7 point Likert scale was used for each painting, the minimum visual art score possible for each respondent was 40 and the maximum score possible was 280.

**Data analysis.** We used Cronbach’s alpha on the visual art and biosocial items to determine internal consistency reliability. We removed items which reduced consistency until α > 0.7 was achieved for each scale. Art items 2 and 4 reduced visual Art scale alpha considerably, while all other visual art items raised alpha. Thus, we eliminated these two items before regression analysis. We retained all six biosocial items. Multiple regression analysis was then run to determine associations between JSE score and age, gender, class year, biosocial score, and visual art score.

## Results/Analysis

71 surveys were collected (see Table 2).

**Table 2.** Study Participant Characteristics

		MS1	MS2	Overall
**Participant Characteristics**	Participants	32	39	71
	Female Participants (%)	18 (56)	20 (51)	38 (54)
	Age (years, mean)	24.1	25.2	24.7
	Age (years, S.D.)	1.86	2.73	2.43
**Art Response Data**	Mean Score	162.7	168.8	166.1
	Median Score	155	164	164
	Score S.D.	32.18	30.36	31.12
	Cronbach’s Alpha	0.71	0.74	0.73
**BioSocial Response Data**	Mean Score	13.7	12.8	13.2
	Median Score	13	12.5	12.5
	Score S.D.	4.47	4.42	4.43
	Cronbach’s Alpha	0.71	0.72	0.71
**JSE Response Data**	Mean Score	119.5	117.3	118.3
	Median Score	120	116	117
	Score S.D.	10.58	11.80	11.24

Cronbach’s alpha for the combined data was 0.73 for the visual art scale and 0.71 for the Biosocial scale. Controlling for class year, age, gender, and Biosocial score, there was a significant positive association between the Art scale and JSE scores (B [95% CI]=0.11 [0.02, 0.19], p=0.014) (see Table 3). Controlling for class year, age, gender, and Art score, there was a significant positive association between the Biosocial scale and JSE scores (B [95% CI]=0.75 [0.16, 1.34], p=0.015) (see Table 3).

**Table 3.** Multiple Regression Analysis Results

			95% CI		
	*Coefficient*	*Standard Error*	*LL*	*UL*	*p-Value*
**Intercept**	108.15	14.01	80.19	136.12	0.000 *
**Art**	0.10	0.04	0.02	0.19	0.014 *
**Biosocial**	0.75	0.30	0.15	1.36	0.015 *
**Age**	-0.61	0.57	-1.74	0.52	0.288
**Year**	-1.45	2.64	-6.72	3.83	0.586

* p < 0.05

There was no significant association between class year, age, or gender and JSE score. The Art and Biosocial scales were not significantly correlated.

## Discussion

Although long recognized as being important to many areas of medicine, empathy is a notoriously difficult quality to measure objectively and reliably, especially over time ( Hemmerdinger, Stoddart and Lilford, 2007; Pederson, 2009). Even the term “empathy” is not well-defined in much of the published research on the subject (Pederson, 2009). Hojat, architect of the Jefferson Scale of Empathy (JSE), described empathy in the medical professions as comprising three meaningful factors: perspective taking, compassionate care, and standing in the patient’s shoes ( Hojat *et al*., 2002a; Hojat, 2016). This research focused on the importance of being curious, rejecting simplicity, going deeper, and acknowledging an emotional dimension to the process. One could argue that the questions we asked students provide the means by which Hojat’s factors can be achieved in the clinical setting.

No other study of empathy has examined visual methods of evaluation, nor have others directly compared results across tests of empathy with the same study cohort. By showing a positive, significant association with a known scale of empathy which is well-validated, we believe we are providing evidence that this visual method of evaluation holds merit for further study. Furthermore, we believe that by using visual art as a proxy for patient stimuli, it may be less susceptible to social desirability bias than other scales of empathy which use crafted textual descriptions of patient-physician interactions. Of note, several study participants afterwards gave feedback that they could not determine the “correct” way to answer an item within the visual art test, but could do so with the JSE easily. Hojat (2016) explicitly states the JSE must not be given in a competitive setting for this very reason.

While there was a significant relationship between the Art scale and the JSE, there was not a one-to-one correspondence. This suggests that the two scales are measuring related but not identical qualities. The questions used in this study emphasized curiosity (#1), emotional connection (#3), nuance (#5), and complexity (#6). Clearly perspective-taking and standing in the patient’s shoes (dimensions of the JSE) require some form of curiosity, nonjudgmentalness, awareness of complexity, and a desire to learn more. But these may better be conceived of as concepts that are in relation rather than synonymous; and the precise nature of their relationships to each other and toward clinical empathy remains to be elucidated.

Further, the JSE is not designed to measure emotional attitudes such as fellow feeling ( Smith, 2017). Compassionate care may or may not require the individual to be “moved” by the suffering of another (it might only require *cognitive understanding* that the other is suffering). Some scholars have posited a model of compassionate empathy in which both cognitive and affective dimensions contribute to caring outcomes, and which they regard as preferable to a more detached clinical relationship ( Post *et al.,* 2014; Jeffrey, 2016). This model has received support from fMRI findings and neuroscience research ( Preusche and Lamm, 2016) but requires further empirical validation ( Ekman and Krasner, 2017).

Additionally, the positive and significant association between the Biosocial scale and JSE raises the question: How do trying circumstances early in life impact empathetic response in medical students and physicians? Our preliminary findings suggest a relationship between experiencing personal difficulties and greater empathy in medical students, confirming existing research with non-health professionals ( Kraus, Côté and Keltner, 2010; Piff *et al.,* 2010). However, similar to the Art scale, the regression analysis results suggest that difficult life experiences are related to empathy as measured by the JSE but are not identical to the constructs in this instrument. Many confounders exist. For instance, because our study participants were high achieving medical students who had overcome early life stress, it may be that they possessed some intrinsic factor that protects or enhances their sense of empathy.

The strengths of association reported between the Art scale and JSE and the Biosocial scale and JSE tell us that these scales are not a substitute for JSE, but that each measures, likely independently, an underlying construct that in some way overlaps with JSE. Thus, what we learned in this study is that, on average, medical students whose responses to art indicated greater curiosity, emotional connection and appreciation for nuance and complexity also tended to exhibit higher levels of empathy. Similarly, medical students who had more stressful life experiences also tended to show higher levels of empathy. The lack of relationship between the Art and the Biosocial scales might be explained by the premise that each is associated with a different aspect of empathy, both of which are partially represented in the JSE. That there appear to be independent effects of Biosocial and Art scales in relation to JSE suggests that the association of higher Art and JSE scores is not explained by these students having had different life experiences.

**Limitations.** This was a single institution study, and medical students at other institutions might show different outcomes. The study design used the JSE as a comparator measure and treated it as the “gold standard” in representing true levels of empathy. However, being in different modalities (one visual, one text-based), the JSE and visual Art scale may only be associated insofar as they overlap in capturing subject empathy via their different methods. We must also consider that there was lack of replication of the Art scale, for which we were only able to establish internal reliability and face validity. This poses the interesting question of whether other art selected would have a similar outcome. Further research is needed to refine and validate the Art scale and to determine the usefulness of this instrument in reliably and accurate assessing medical student empathy. For example, an interventional study could investigate the educability of student empathy (and what we have proposed as related qualities of curiosity, connection, nuance, and complexity) through exposure to art. As well, the precise nature of “exposure to art” needs to be studied in more detail, including assessing the significance of such variables as background of instructor, method and length of instruction, type of art (e.g., representational, abstract, classic, contemporary etc.), and variations in setting (e.g., museum vs. classroom) and learners (i.e., homogenous [medical students only] or interprofessional [nursing and medical students]). It should be remembered that this study was not designed to attempt conclusive answers. Rather, its purpose was to explore several previously unexamined aspects of medical student empathy.

## Conclusion

Both the de novo visual Art and Biosocial scales achieved significant positive associations with the JSE, a benchmark measure of physician empathy. They additionally both achieved good internal consistency, indicating scale items were measuring similar respondent qualities. These results support the study hypotheses that response to visual stimuli, as well as economic and experiential formative factors, are each independently associated with empathy.

## Take Home Messages

• Clinical empathy is an important quality to identify in future physicians.
• Standard measures may not fully assess important dimensions of the construct of empathy.
• Medical students’ interpretation of visual art may provide information about empathy-related qualities of curiosity, emotional, connection, nuance, and complexity.
• Early stressful life events may be related to greater empathy in medical students.
• More research is needed to explicate relationships between empathy, visual art interpretation, and stressful life events.

## Notes On Contributors

Sam Sampson PGYI is a registered respiratory therapist with six years’ experience in critical care medicine, and is doing a residency in diagnostic radiology at Kaiser Permanente Los Angeles Medical Center.

Johanna Shapiro is a Professor in the Department of Family Medicine and Director of the Program in Medical Humanities & Arts, University of California Irvine, School of Medicine, Orange, CA, USA.

John Billimek is Assistant Professor in Residence at the Health Policy Research Institute, the Division of General Internal Medicine, the Department of Family Medicine and the Program in Medical Education for the Latino Community (PRIME-LC), UC Irvine, School of Medicine, Orange, CA, USA.

Joel Shallit is an Adjunct Assistant Professor in the Department of Family Medicine, UC Irvine, School of Medicine, Orange, CA, USA.

Julie Youm is a Clinical Assistant Professor in the Department of Emergency Medicine, and Assistant Dean, Education Compliance and Quality, UC Irvine, School of Medicine, Orange, CA, USA.
