# Supplementary material for: Medical Student Interpretation of Visual Art: Who's Got Empathy?
Source: MedEdPublish (2016). 2018 Oct 17;7:206. Originally published 2018 Sep 11. [Version 2] doi: 10.15694/mep.2018.0000206.2 (PMC10701853; doi:10.15694/mep.2018.0000206.2)
Supplement: Peer review history

# Reviewer report: Reviewer response for version 2

**DOI:** 10.21956/mep.20228.r31335

**Published:** 2019-04-30

**Authors:** Saee Deshpande

**Affiliations:** 1 Maharashtra University of Health Sciences, Nashik- VSPM Dental College Nagpur

This review has been migrated. The reviewer awarded 4 stars out of 5 A very important topic of empathy dealt in systematic way.visual method needs to be further investigated to ascertain its merit.the paper is quite useful for those involved in medical humanities.

Reviewer Expertise:

NA

No decision status is available

# Reviewer report: Reviewer response for version 2

**DOI:** 10.21956/mep.20228.r31336

**Published:** 2019-01-13

**Authors:** Susmita Reddy Karri

**Affiliations:** 1 Topiwala national medical college and BYL nair charitable hospital, mumbai. india

This review has been migrated. The reviewer awarded 5 stars out of 5 An extremely interesting paper which focuses on the difficult but often spoken topic of empathy.

Reviewer Expertise:

NA

No decision status is available

# Reviewer report: Reviewer response for version 2

**DOI:** 10.21956/mep.20228.r31338

**Published:** 2018-10-18

**Authors:** Margaret Chisolm

**Affiliations:** 1 Johns Hopkins University School of Medicine

This review has been migrated. The reviewer awarded 5 stars out of 5 A great paper, made even better by recommendations to help guide future research on this important topic, such as study of variables such as background such as curriculum duration, methods, setting, and targeted learners.

Reviewer Expertise:

NA

No decision status is available

# Reviewer report: Reviewer response for version 2

**DOI:** 10.21956/mep.20228.r31337

**Published:** 2018-10-18

**Authors:** Trevor Gibbs

**Affiliations:** 1 AMEE

This review has been migrated. The reviewer awarded 5 stars out of 5 Thank you for re-submitting this paper although I feel that the paper did not require that much improvement. The improvements that the authors have made however, further improve what was already a very good paper. The expansion and explanation of the ART scale and the addition of comments on the where to now element of the paper certainly add to the papers value. This is certainly a very interesting and important paper for all to read, especially those concerned with the Humanities in healthcare education and the difficult subject of empathy- very well done.

Reviewer Expertise:

NA

No decision status is available

# Reviewer report: Reviewer response for version 1

**DOI:** 10.21956/mep.18984.r27207

**Published:** 2018-09-26

**Authors:** Margaret Chisolm

**Affiliations:** 1 Johns Hopkins University School of Medicine

This review has been migrated. The reviewer awarded 5 stars out of 5 This is a great topic. The authors articulated the context for the study very well. It was refreshing to see the use of art tied to a learner outcome (empathy) known to affect patient outcomes; and I was especially impressed by the use of the JSE and development/use of the Art scale, the limitations of which are clearly articulated. I look forward to learning more about the Art scale and seeing it further refined and validated. The results raise two particularly interesting questions: 1) can curiosity, emotional, connection, nuance, and complexity, as measured by the Art scale, be taught? and 2) if so, does this result in increased empathy? Also, I would be interested in knowing if there are differing effects of museum-based art viewing experiences versus viewing photographs of the art. Lots of future work to be done, but this is a great start to an important area of inquiry of interest to all healthcare profession educators.

Reviewer Expertise:

NA

No decision status is available

# Reviewer report: Reviewer response for version 1

**DOI:** 10.21956/mep.18984.r27206

**Published:** 2018-09-12

**Authors:** P Ravi Shankar

**Affiliations:** 1 American International Medical University

This review has been migrated. The reviewer awarded 5 stars out of 5 I enjoyed reading this interesting paper. Empathy is very important in healthcare practice and is a concept which is difficult to define. We start our medical humanities module with a session on empathy. I and my colleagues had studied empathy among students using the Jefferson scale of physician empathy. I do agree that it concentrates more on the cognitive aspects of empathy. Other instruments looking at other dimensions of empathy may be required. The authors examine two instruments created by them to study empathy, the biosocial scale and the visual art empathy measure and compare these to the Jefferson scale which is accepted as a standard measure of empathy. I would be interested in obtaining more information about the biosocial scale. The visual art empathy measure seems very interesting. How did the authors decide on which ten paintings to include to measure empathy? The authors’ work opens the way to examine other measures of empathy in the medical profession. The article will be of interest to all educators with an interest in the humanities.

Reviewer Expertise:

NA

No decision status is available

# Reviewer report: Reviewer response for version 1

**DOI:** 10.21956/mep.18984.r27205

**Published:** 2018-09-11

**Authors:** Trevor Gibbs

**Affiliations:** 1 AMEE

This review has been migrated. The reviewer awarded 5 stars out of 5 I really enjoyed reading this paper about the very important topic of empathy and its teaching. I felt that the paper was well researched ( a very good list of important papers on the topic), was very well explained and had a very helpful flow to it. There were limitations to the paper which the authors explored and I can see their way forward very clearly. I learned from this paper, particularly about the relationship between social background and life events and later showing the qualities of empathy- I could immediately think of a piece of research in my own field with developing countries and this.I would strongly recommend this paper to all those working in curricula development and the Humanities- very well done

Reviewer Expertise:

NA

No decision status is available
